# Some dogs can find the payoff-dominant outcome in the Assurance game

**DOI:** 10.1016/j.isci.2023.108698

**Published:** 2023-12-09

**Authors:** Mayte Martínez, Selina Schöndorfer, Lauren M. Robinson, Sarah F. Brosnan, Friederike Range

**Affiliations:** 1 Domestication Lab, Konrad Lorenz Institute of Ethology, University of Veterinary Medicine Vienna, Vienna 1160, Austria; 2 Language Research Center, Georgia State University, Atlanta, GA 30303, USA; 3 Department of Cognitive Biology, University of Vienna, Vienna 1030, Austria; 4 Department of Psychology, University of Michigan, Ann Arbor, MI 48108, USA; 5 Departments of Psychology and Philosophy, Neuroscience Institute, Center for Behavioral Neuroscience, Georgia State University, Atlanta, GA 30303, USA

**Keywords:** Canine behavior, Biological sciences, Zoology, Cognitive neuroscience

## Abstract

Studies on coordination often present animals with the choice of either cooperating or remaining inactive; however, in nature, animals may also choose to act alone. This can be modeled with the Assurance game, an economic game that has recently been used to explore decision-making in primates. We investigated whether dyads of pet dogs coordinate in the Assurance game. Pairs were presented with two alternatives: they could individually solve an apparatus baited with a low-value reward (*Hare*) or they could coordinate to solve a cooperative apparatus baited with a high-value reward for each dog (*Stag*). All individuals matched their partner’s choices, but after controlling for side bias, only four out of eleven dyads consistently coordinated on the payoff-dominant strategy (*Stag-Stag*). Thus, some dogs are capable of finding coordinated outcomes, as do primates, at least when their partner’s actions are visible and coordination results in the biggest payoff for both individuals.

## Highlights

• We used economic games to study decision-making in dog-dog coordination
• Pet dogs coordinated with their partner’s choices in the Assurance game
• Four out of eleven dyads consistently coordinated on Stag, like some primates do
• This payoff-dominant outcome is likely achieved with simple mechanisms

## Graphical abstract

**Figure:**
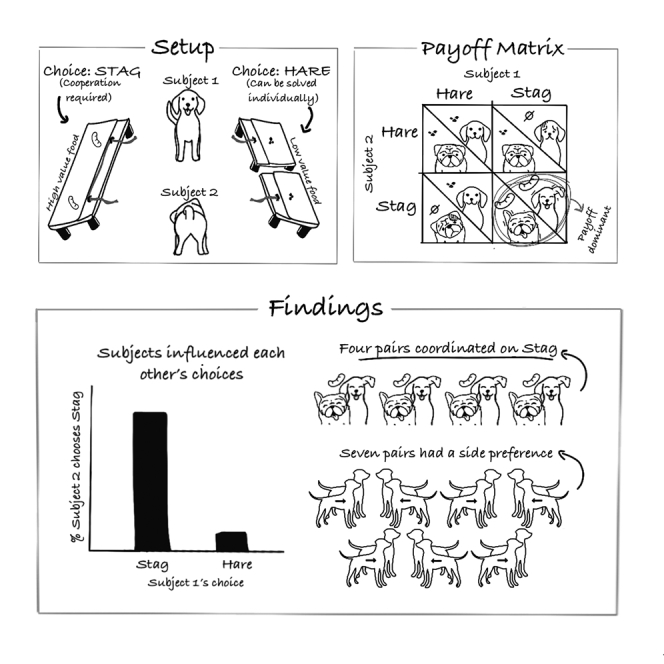

## Introduction

Coordination among group-living animals often occurs in cooperative contexts (e.g., hunting, parental care), in which coordination benefits all participants.^1^ When subjects’ objectives are aligned, coordination may arise due to subjects independently acting toward the same goal (e.g., by-product mutualism^2^), without understanding the cooperative situation.^3^ Thus, a key question is to what extent animals consider their partner’s actions in the task and use that knowledge to adjust their own actions accordingly.^4^

Among animals, humans’ unique flexibility in cooperating with others is thought to be grounded in their ability to recognize the role and intentions of their partners. Therefore, studying the degree to which other animals recognize the consequences and the importance of their partner’s decisions during coordination is crucial to shed light on the evolutionary origins of human cooperation.

Most research exploring how non-human animals coordinate actions with each other has relied on the cooperative pulling/pushing paradigm. In this paradigm, two or more individuals must simultaneously pull handles/ropes,^5^^,^^6^^,^^7^^,^^8^^,^^9^ or press buttons,^10^^,^^11^^,^^12^ to gain access to food rewards for both individuals. Thanks to these studies, we know that some species understand at minimum that they need a partner present to solve the task.^13^ However, one disadvantage of the cooperative pulling/pushing tasks is that there is only one option to access the rewards: pulling/pushing at the same time as their partner. If subjects fail to do that, they obtain nothing. This does not represent the complexity of options that animals have in nature, wherein animals frequently face problems that can be solved either individually or cooperatively. For instance, an animal might choose to participate in group-hunting to obtain a high-quality food or they may prefer to avoid the risk that comes from working with others (e.g., their partner can monopolize the rewards^14^^,^^15^) and instead choose to forage alone for lower-quality food. To explore whether subjects understand the role of their partners in such scenarios, a different task is required. Economic games offer a promising approach that overcomes the limitations of previous paradigms to model a wider range of situations.

Games derived from experimental economics are highly structured tasks that represent complex social situations in the form of simple, usually dichotomous, choices and in which outcomes are dependent on the combination of the participants’ choices.^16^ Economic games have been historically used to study the adaptative function and evolution of cooperation.^17^ However, in recent times they have also proven fruitful for uncovering the cognitive mechanisms, particularly from a comparative perspective, due to the simplicity and flexibility of these games.^18^ Game’s choices can be implemented in diverse ways and adapted to each species, using, for instance, tokens,^19^ trays,^20^ or computer icons.^21^ Specifically, the decision to hunt together for a high-quality reward or forage alone for a lesser reward can be modeled using the Assurance game.

In the Assurance game, or Stag Hunt game, two individuals must choose one of two options: *Stag* or *Hare*. The payoff matrices for the Assurance game differ across disciplines and studies; however, they maintain key features that make the game a coordination game. It is always the case that if both players choose *Stag*, they both obtain the highest payoff, and mutual *Hare* results in a lower payoff for everyone. Uncoordinated choices (one individual chooses *Hare* and another individual chooses *Stag*) result in no reward for the individual who chooses *Stag* and a reward for the individual who chooses *Hare* that is smaller than the *Stag-Stag* payoff.^22^

In the Assurance game, coordination on *Stag* represents the payoff-dominant Nash-Equilibrium (NE), the strategy that results in the highest payoff for both players. Although players maximize their benefits by coordinating on *Stag*, choosing *Stag* can be risky, as selecting this option when the partner chooses *Hare* results in no payoff. Choosing *Hare* is the riskless choice, as it always results in some reward.

Five non-human primate species have been tested in the Assurance game so far: squirrel monkeys,^23^ capuchin monkeys, rhesus macaques, chimpanzees, and humans.^21^^,^^24^ Every species but squirrel monkeys eventually found the payoff-dominant NE, reflecting the species’ ecology: references documenting cooperation among squirrel monkeys in the wild are very rare,^25^ whereas it is common in the other tested species.^3^^,^^26^^,^^27^ Additionally, researchers have used variations of the game to investigate decision-making strategies. For instance, the performance of chimpanzees hinged on their experimental history; only subjects with extensive experience in cognitive testing found the payoff-dominant NE. These individuals maintained coordination regardless of whether they could see their partner’s choices and quickly adjusted to a new setup (different tokens), which suggests they understood the payoff matrix.^24^^,^^28^ Other research using a different task with the same payoff structure shows that chimpanzees’ performance is influenced by leader-follower dynamics, with one individual consistently initiating movement toward the *Stag* option and the other following.^29^^,^^30^ Capuchin monkeys solved the task by matching each other’s choices but failed when partner’s choices were not visible.^21^^,^^24^^,^^31^ Finally, rhesus macaques found the payoff-dominant NE independently of the visibility of the partner’s choice.^21^ In a follow-up study,^32^ humans and rhesus monkeys played the Assurance game with a computer-simulated partner (an algorithm varying *Stag* play frequency between 0% and 100%); only humans matched the proportion of *Stag* choices of the simulation. Rhesus macaques showed a strong *Stag* bias, independently of the simulation’s strategy. This reveals that a preference for the on-average higher paying option (*Stag*) is driving the macaques’ choices.

Although these studies suggest that species play the game differently, further research is needed to shed light on whether animals recognize that the decisions taken by their partners affect the outcomes in these games. Reaching that goal requires comparing the performance of species from very different taxa,^33^^,^^34^ but so far most of the research on the Assurance game and other economic games has focused on non-human primates. Additionally, the relative simplicity of the Assurance game makes it a valuable tool to study species untested in this type of economic game. Here, we aim to fill this gap by using the Assurance game to test a non-primate species: dogs (*Canis lupus familiaris*). It has been hypothesized that domestication has provided dogs with human-like social cognitive abilities.^35^^,^^36^^,^^37^ According to this idea, dogs might be a telling model species regarding the evolution of human cooperation^38^ and reciprocity.^39^^,^^40^ Thus, testing both primates and dogs using similar paradigms can provide evolutionary insights that may not be possible to obtain from studying primates alone. Dogs are also a good choice in their own right, as they are skilled at understanding human communication^41^^,^^42^^,^^43^^,^^44^ (but see^45^), even outperforming non-human primates.^46^^,^^47^ In the cooperative pulling/pushing paradigm, dogs can coordinate with humans,^48^ waiting for and recruiting human partners when needed.^49^ Further research suggested that dog-human coordination might rely on leader-follower dynamics, with dogs following human signals.^12^^,^^50^

Intriguingly, however, the social skills that dogs display with human partners do not seem to generalize to interactions with conspecifics, and pairs of dogs systematically fail to coordinate their actions in a cooperative pulling task.^6^^,^^51^ Dogs’ inability to manipulate the apparatus simultaneously might stem from either a poor understanding of the cooperative situation or a lack of tolerance around food sources.^52^^,^^53^ Thus, more research is needed to understand whether and how dogs coordinate with conspecifics. Here, we set out to test pet dogs on the Assurance game. We limit our sample to pet dogs living in the same household, to explore their decision-making unhindered by tolerance issues. Nevertheless, we still expected some variability in tolerance among dyads, which can influence their coordination with their partner.^5^^,^^7^^,^^9^^,^^15^ To account for this, we tested the dyads’ tolerance in a food context before the Assurance game.

**Figure 1:**
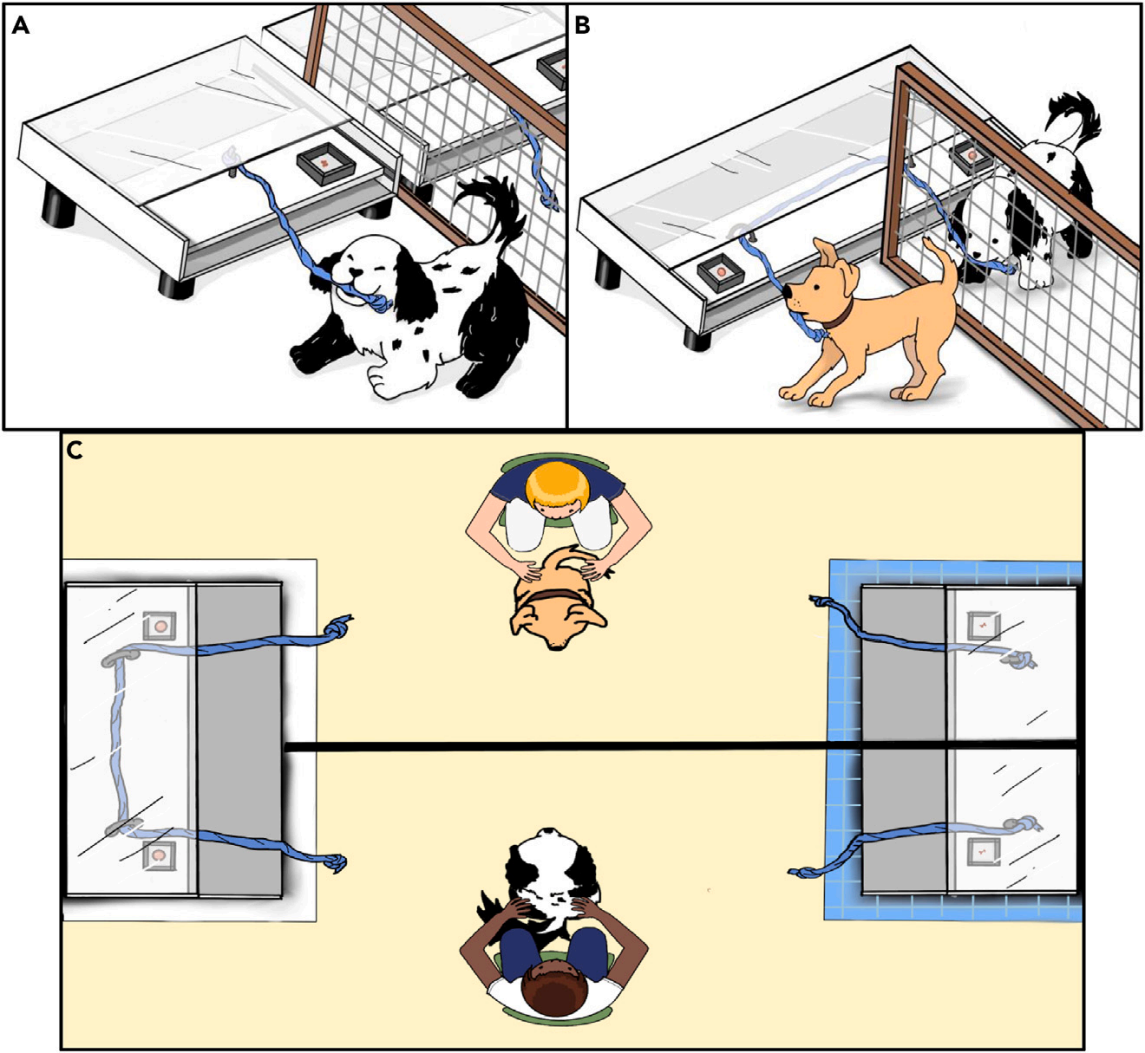
Setup for the Assurance game (A–C) *Hare* table (A), *Stag* table (B), and starting position (C). In the game trials, subjects can choose whether they want to cooperate (*Stag*) or defect (*Hare*) by choosing whether to pull the rope from one or another sliding table. (C) The *Stag* table is on the left and the *Hare* table is on the right.

Our setup for the Assurance game had the form of a foraging task (similar to^29^^,^^30^, see Figure 1), in which dogs had to choose between an apparatus baited with a low-value reward that they could solve individually (*Hare*) and a cooperative apparatus baited with a high-value reward that required the two animals to coordinate to obtain the rewards (*Stag*) (see payoff matrix in supplemental information, Figure S1). The *Stag* apparatus was a cooperative pulling table, as in the cooperative pulling paradigm. With the inclusion of the individual apparatus, we overcome one of the main limitations of the cooperative pulling/pushing paradigm: the absence of an alternative choice that could potentially result in a reward. To control for spatial preferences, we conducted two sessions, exchanging the placement of the *Stag* and *Hare* apparatus in the second session. Because we do not know which strategy dogs may use in the Assurance game, we implemented the game in a way that the partner’s choice was always visible to the subjects. Each member of the dyad was assigned the role of Subject 1 (S1) or Subject 2 (S2). Because S2 was always released 1 s after S1, S2 could see S1’s movements before making its own choice. That way dogs could succeed even following a simple matching strategy, as was observed in the capuchin monkeys,^21^^,^^24^ or if they prefer the highest paying option (*Stag*), as was observed in rhesus monkeys.^32^ Hence, the performance in the game itself is not enough to determine whether they recognize their partners as a social agent whose decisions have consequences in the game’s outcomes. To tackle this question, we tested whether coordination during the Assurance game influenced the subject’s behavior toward their partner after the game. Brucks and colleagues^54^ used this method, showing that dogs reduced contact-seeking toward their conspecific partner and the experimenter after receiving a disadvantageous payoff. Accordingly, in addition to measuring the tolerance levels in food context before the Assurance game, we repeated the tolerance tests after the Assurance game. We predicted that if dogs understood that their outcomes were contingent on their partner's choices, they would show increased tolerance (measured as cofeeding time) toward their partner after mutual Stag play in the Assurance game.

## Results

### Do dogs find the high payoff equilibrium (*Stag-Stag*) in the Assurance game?

**Figure 2:**
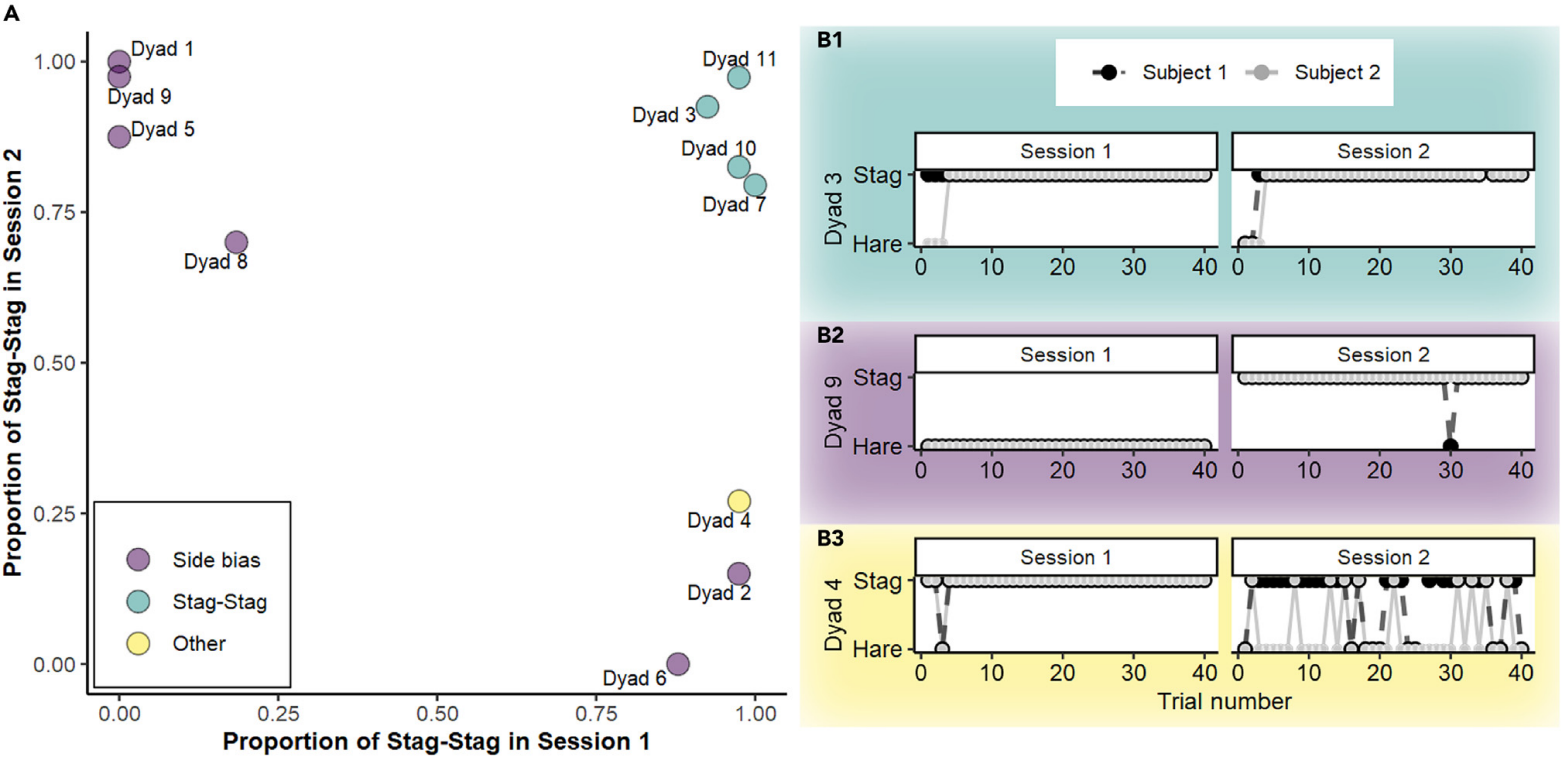
Dyads’ performance in the Assurance game (A) 2D scatterplot showing the proportion of Stag-Stag choices by dyad (excluding trials in which either S1 or S2 did not make a choice), in session 1 (x axis) versus session 2 (y axis). Dyads that showed a majority of *Stag-Stag* choices in both sessions are shown in green. Dyads that showed a majority of choices of *Stag* in one session and *Hare* in the other session (side bias) are shown in purple. The dyad that coordinated on Stag in the first session and made uncoordinated choices in the second session (other) is shown in yellow. (B) Examples of dyads’ performance in the Assurance game. Each dot represents S1’s (black) or S2’s (gray) choice in each trial. Black dots are bigger than gray dots, and thus, when choices overlap and the gray dot is on the top of the black dot, the graph shows the gray dot with a black ring around it. Blank spaces indicate trials in which the individual did not make any choice. For other dyads, see Figures S6–S8.

Comparing the proportion of each possible outcome (*Stag-Stag*, *Hare-Hare*, *Hare-Sag*, and *Stag-Hare*) with chance level (25%) by means of chi-square goodness-of-fit test, we found that all dyad’s performances deviated from chance (see Figure 2; Table S3; Figures S6–S8). By inspecting the standardized residuals of each outcome^55^ (significant at p < 0.01 for +/− 2.58), we determined the most frequent outcome for each dyad and session and whether it deviated from chance. Four of the dyads (dyads 3, 7, 10, and 11) consistently coordinated on *Stag* in both sessions, whereas the outcomes of the other dyads could be explained by at least one of the members of the dyad showing an individual preference for one side of the room or, in the case of dyad 4, perhaps tolerance issues. Specifically, six dyads changed their preference when we changed the position of the *Hare* and the *Stag* tables in the second session (i.e., in both sessions they always chose the same side, independently of the table that was placed there): four of them showed a significant preference for *Hare-Hare* in the first session and changed to *Stag-Stag* in the second session (dyads 1, 5, 8, and 9), and two of them chose *Stag-Stag* in the first session and switched their choice, showing a majority of *Hare-Hare* in the second session (dyads 2 and 6). Finally, dyad 4 coordinated on *Stag* in the first session and made uncoordinated choices in the second session, with most trials in which S1 chose *Hare* and S2 chose *Stag*. Importantly, uncoordinated choices in this dyad started after the only aggressive interaction that we observed during the Assurance game, in which S1 growled at S2.

Inspecting different factors that could have influenced whether dyads coordinated on *Stag*, we found that *Stag-Stag* choices were not more likely to happen in the last trials or in the second session and that neither tolerance (cofeeding in the tolerance tests) nor S1 and S2’s age have an effect on whether dogs coordinate in the payoff-dominant NE (*Stag-Stag model*, full-null model comparison: *χ2* = 10.41, *df* = 15, p = 0.79, see supplemental information, Table S4).

### Do dogs flexibly adjust to their partner’s choices in the Assurance game?

**Figure 3:**
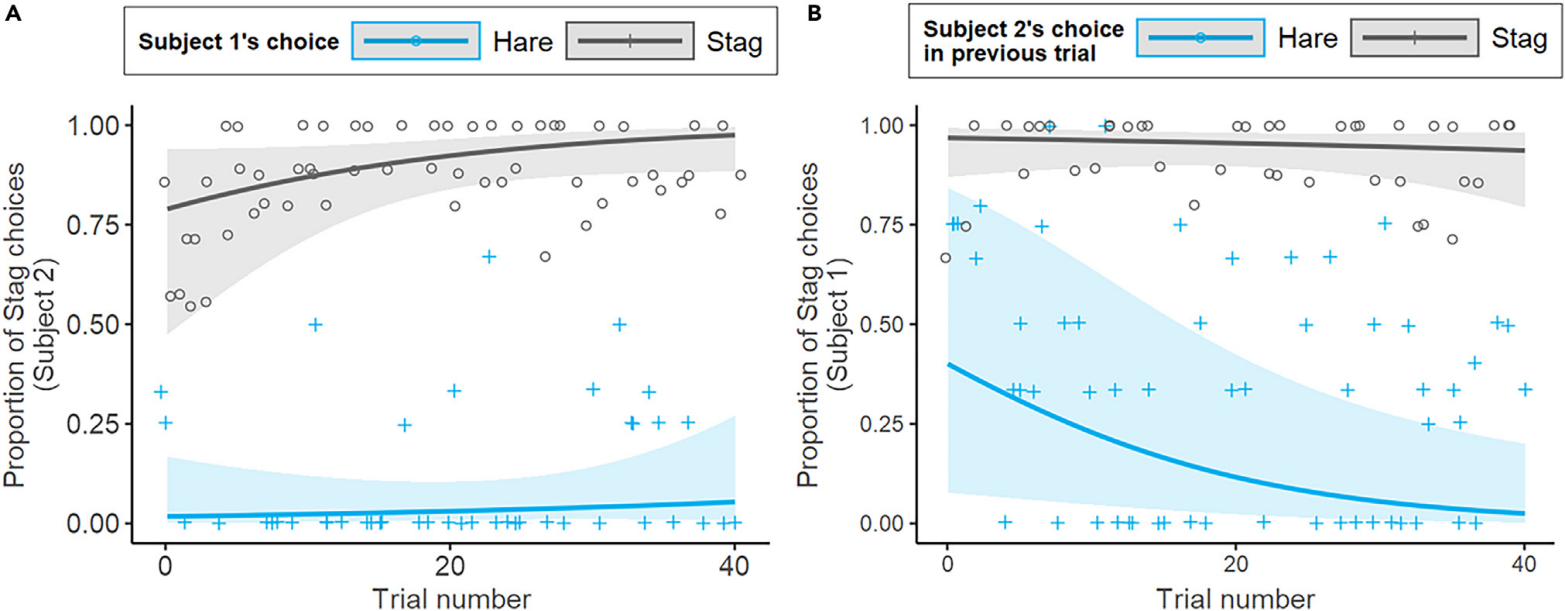
Relationship between S1 and S2’s choices Proportion of *Stag* choices of S2 depending on S1’s choice (A) and proportion of *Stag* choices of S1 depending on S2’s choice in the previous trial (B) (11 dyads). Lines represent the fitted model for the effect of trial number and S1/S2 choices (blue lines represent the predicted response when the other individual choice is *Hare*; gray lines represent the predicted response if the other individual chooses *Stag*). Dots represent the proportion of *Stag* choices of S2 (left) and S1 (right) averaged by session, trial, and whether the other individual choice is *Hare* (circular points) or *Stag* (cross-shaped points). Shadowed area corresponds to 95% Wald confidence intervals.

In the *S2 model*, we found that Subject 2 was more likely to choose *Stag* when S1 also made that choice (full-null model comparison: *χ2* = 23.31, *df* = 4, p < 0.01; estimate for the effect of S1’s choice = 5.947, *SE* = 0.770, p < 0.001; see Figure 3A). None of the interactions between the predictor (S1’s choice) and the control variables was significant (see Table S5), showing that the effect of S1’s choice on S2’s choice did not depend on trial number, or whether the previous trial resulted in a reward, or on dyad type (i.e., it was the same in dyads that showed little variability in their responses—mostly coordinated on *Stag*- and the rest of the dyads).

In turn, the *S1 model* revealed that Subject 1 was more likely to choose *Stag* if S2 had chosen *Stag* in the previous trial (full-null model comparison: *χ2* = 13.97, *df* = 3, p = 0.003; estimate for the effect of S1’s choice = 5.10, *SE* = 1.24, p = 0.045; see Figure 3B). This effect did not depend on trial number or dyad type (none of the interactions in the model were significant, see Table S6). We did find a main effect of dyad type (estimate = 6.22, *SE* = 2.13, p < 0.001), meaning that S1 chose *Stag* more often in dyads that consistently coordinated on *Stag* than in the other dyads.

### Do dogs change their tolerance toward their partners after successful coordination in the Assurance game?

The interaction between HVR consumed and condition (training session/Assurance game) was significant (*Tolerance model*, full-null model comparison: *χ2* = 4.19, *df* = 1, p = 0.041), indicating that dogs spent more time cofeeding if they had eaten more HVR before the tolerance test (that is, more coordination on *Stag* resulted in more time cofeeding in the tolerance tests), but only when that food was obtained in the Assurance game (see supplemental information, Figure S9). Regardless, we found that the estimate of this effect was not stable (see supplemental information, Table S7), which indicates that the effect might be limited to some dyads. Visual inspection of the results revealed that only two dyads showed an increase in cofeeding after more coordination in the Assurance game (dyads 1 and 3, see Figure S10). Overall, the effect of the interaction that we found is not reliable, which may be due to our small sample size, and we might find a different effect in either direction (smaller or stronger) with a different sample.

## Discussion

Comparative work using the Assurance game in primates showed that at least some pairs of all species were capable of coordinating on mutually beneficial outcomes, but the mechanism underlying coordination varied across species and even groups.^13^^,^^18^^,^^56^ In the current study, we demonstrated that some pet dog dyads also coordinated their choices on the payoff-dominant NE, while the remaining dyads changed their preference depending on the session. Importantly, we found that dogs adjusted their choices based upon their partner's choices, even when they did not coordinate on *Stag*. Whereas this could be achieved by simple mechanisms, such as matching (a mechanism seen in some primates), we cannot rule out that they understood their partner’s role. A more in-depth analysis of our findings is presented in the following section.

Not every pair coordinated: of eleven dyads of dogs, four of them coordinated their choices on *Stag*, the payoff-dominant NE, in both sessions, which maximized both dogs’ rewards. Subjects also adjusted their choices around their partners; when we explored how the choices of the partners affected each other, we found that S2 was more likely to choose *Stag* when S1 chose *Stag*, and S1 was also more likely to choose *Stag* if S2 had chosen *Stag* in the previous trial. This is reminiscent of macaques and capuchins in Prisoner’s dilemmas, who also change their choices based on those their partners make.^31^^,^^57^ The model results were the same for the dyads that found the payoff-dominant NE and for the rest of the dyads, suggesting that they pay attention to one another’s choices no matter what the outcome/strategy they choose.

These findings stand in contrast with previous research in which pairs of dogs failed in a cooperative pulling task^6^ and showed only a moderate improvement after extensive training.^51^ The difference is striking because the apparatus was the same as our *Stag* table; in those studies,^6^^,^^51^ dogs were required to pull a rope at the same time on the same table to get the food. There was, however, one key difference; their sample was composed of pack-living dogs, and, as they argued, lack of tolerance, rather than cognitive limitations, can explain why dogs were reluctant to act simultaneously. We pre-selected tolerant dyads by limiting our sample to pet dog pairs living in the same household, a selection bias that undoubtedly influenced our results (cf.^58^). This may be why, contrary to studies suggesting that affiliation and tolerance are key to coordination,^9^^,^^15^^,^^59^ we found no relationship between tolerance around food sources and the proportion of *Stag-Stag* choices of each dyad. Moreover, two elements of our setup might have reduced the necessity for tolerance. First, owners were in the room for the whole duration of the experiment, which presumably helped to inhibit conflicts; and second, dogs were separated by a fence, preventing physical contact between them. In this regard, we observed one aggressive interaction in dyad 4, which appeared to lead to uncoordinated choices. This is in line with previous work that has found that when conflicts arise, dogs use an avoidance strategy that can easily lead to the breakdown of cooperation.^6^^,^^53^ Thus, future work with more variable relationship quality might find an effect.

Six dyads preferentially choose *Stag-Stag* in one session and *Hare-Hare* in the other session, which indicates either a side bias in both individuals (both prefer the same side of the room, without attending to their partner’s choices) or that one of the individuals was side biased and the other individual followed them. Indeed, if one of the players in the Assurance game invariably chose the same option, the best-paying possibility for the second player would be to make the same choice. The dogs do appear to be taking each other’s choices into account; our analyses revealed that S1 was more likely to choose *Hare* if S2 chose *Hare* (instead of *Stag*) in the previous trial.

When considering all the aforementioned factors, there are two potential (and not mutually exclusive) strategies in play. First, the four dyads that found the payoff-dominant NE seem to understand the payoff matrix, showing a majority of *Stag*-*Stag* choices (although sometimes, dogs would also choose *Hare-Hare*). Second was matching, which does not always yield the highest payoff, but can be the best solution with a partner who does not understand the task or shows a side bias. This strategy has been seen in some primates; capuchins only coordinated when they could see their partner’s choices, suggesting matching,^21^^,^^24^ and in one study, chimpanzees who did not have extensive testing experience used matching (or showed no strategy).^24^ In another study,^60^ macaques played a version of Bach-or-Stravinsky in which the partner’s actions were always visible. In this game, two individuals received a better payoff if their choices were coordinated in either of two options. However, one coordination option provided a higher reward for one individual, whereas the other option provided a higher reward for the second individual. Some pairs of macaques consistently coordinated on just one of the two options, even when the most payoff-equalizing strategy would have been to alternate which option they coordinated on (as most human pairs did in the study), whereas other macaque pairs found an efficient and payoff-equalizing strategy by coordinating on the same side. This evidence, together with our results, suggests that non-human animals tend to rely on matching mechanisms when they can see their partner’s choice.

In fact, imitation is believed to play a crucial role in the evolution of cooperation by shaping behavioral strategies such as Tit-for-Tat, in which subjects imitate their partners’ previous behavior (e.g., whether they receive help from a social partner^61^). Imitation can enable seemingly coordinated behaviors without a real understanding of the payoff matrix when individuals copy the other’s actions, for instance, to obtain a food reward. Future studies should explore whether dogs can maintain coordination when the use of a matching strategy is challenged, such as when their partner’s choices are not visible, or when the choices of *Stag* and *Hare* involve different actions for each subject.

Although we cannot answer the question, it is useful to speculate on what mechanisms may be underlying the dogs’ choices. Dogs’ matching strategy could have emerged through basic mechanisms such as local enhancement^62^ or stimulus enhancement.^63^ From an ecological perspective, these simple rules could explain cooperation among free-ranging dogs, which consists mainly in territorial defense.^64^^,^^65^^,^^66^ Whereas in these situations, in which subjects have access to the partner’s actions, low-level cues might be sufficient to succeed; we cannot exclude that they also consider other aspects of the social situation when making decisions. In this regard, we predicted that if dogs understood their partner's role in the HVR obtained in the Assurance game, the amount of time that they spent cofeeding in the tolerance test would depend on the number of Stag-Stag choices in the Assurance game. That is exactly what our model revealed. Unfortunately, those results, although significant, were very unreliable.

In 4 out of 11 dyads, dogs were able to behaviorally coordinate to obtain a mutually beneficial reward. However, studies in primates suggest higher levels of coordination, such as chimpanzees (8 individuals/12 pairs, average proportion of *Stag-Stag* ± se = 0.91 ± 0.06),^29^ rhesus macaques (8 individuals/5 pairs, average proportion of *Stag-Stag* ± se = 0.81 ± 0.09),^21^ and capuchin monkeys (12 individuals/6 pairs, average proportion of *Stag-Stag* ± se = 0.83 ± 0.13),^31^ but not squirrel monkeys (10 individuals/5 pairs, average proportion of Stag-Stag ±se = 0.33 ± 0.13),^23^ but there are several caveats to consider. First, because our subjects were brought into the laboratory, we were limited in how often we could test them, thus they received only two sessions of 40 trials each (whereas some primates have received up to 10 sessions of 60 trials). With more experience, more dyads might have overcome their side bias and found the payoff-dominant NE. Indeed, the proportion of dog dyads that found the payoff-dominant NE is similar, if not higher, than in primates experiencing the task for the first time. For example, when Brosnan and colleages^24^ first tested chimpanzees and capuchin monkeys, only one out of six capuchin monkey dyads and two out of fourteen chimpanzee dyads found the payoff-dominant NE in the first 10 sessions (with 30 trials per session), with the remaining pairs matching their partner’s choices or showing no identifiable strategy. With more exposure to the game, the same capuchin monkeys coordinated in the payoff-dominant NE.^21^^,^^31^^,^^67^ Thus, even when dogs in our study were tested with a considerably lower number of sessions in comparison with other species, four dyads (36% of our sample) were able to find the payoff-dominant NE.

We tested dogs for the first time in an economic game, aiming to break new ground and propel new research avenues. We encourage other researchers to extend the use of economic games to different populations of dogs, such as free-ranging and pack-raised dogs or dog-dog and dog-human dyads with different degrees of familiarity. This would allow us to better disentangle the effect of social relationships between partners from their understanding of the task contingencies. Additionally, it is yet to be tested whether dogs could find the NE in anti-coordination games that cannot be solved using matching, as players benefit from playing the opposite strategy of their opponents. For example, in the Hawk Dove game, often used in the context of producer-scrounger dynamics and social learning,^68^^,^^69^ players achieve the highest payoff if they defect while their partner chooses cooperation, but if both players defect, that results in the highest cost for both of them. Finally, more research is needed to explore to which extent animals understand the role of their partners when playing economic games. Ideally, that research should be part of a research program that systematically compares, using equivalent procedures, whether and how animals across taxa coordinate and make decisions.^70^^,^^71^ Ultimately, this knowledge will shed light on the evolutionary trajectory of social decision-making.^72^

### Limitations of the study

A crucial constraint in our study is the limited number of dyads that we were able to test due to constraints in recruiting volunteers who had two dogs that passed criterion and were willing to bring them repeatedly to the laboratory. All the pairs were similar in age (all adults), could not mate (for the mixed-sex dyads, no female was in heat and at least one of the two members was neutered), and all dyads were formed by dogs living in the same households and with a tolerant relationship outside the testing environment. However, although we accounted for dyad identity in our model, our sample size did not allow us to fit more complex models encompassing factors such as dyad composition and other potentially influential variables such as subject’s sex. Nonetheless, we did account for variability between dyads by incorporating dyad identity as a random intercept in the models. Perhaps future studies will be able to ask these questions with a larger sample.

Furthermore, in the Assurance game, the *Stag* and *Hare*’s values are typically the same for both participants unless the authors are explicitly testing the role of different values in affecting responses.^67^ In this study, we had to use food individually selected for each dog due to dietary and time limitations. Thus, out of 11 pairs, for three of them the LVR was a different food within a dyad, and for two pairs both the HVR and LVR were different within the dyad. Although this could have influenced their decisions (i.e., by modifying the perceived payoff matrix, for example, if a player considers the *Hare* of the other player as valuable as their own *Stag*), our data suggest that it did not. Considering both HVR and LVR, one of the four pairs that coordinated had different LVR and HVR; considering LVR, two of the four pairs that coordinated had different LVR, and three out of seven that did not coordinate had different LVR. Comparing the dyads that coordinated and the ones that did not, the variation in the proportions of dyads that have the same vs. different types of food does not suggest a big impact. However, our sample size is small to give a clear answer, and future research will hopefully be able to address this issue when considering coordination in dogs.

Finally, our study was focused on the proximate mechanisms of coordination, and future studies should address how those have been selected by natural selection (e.g., kin selection,^73^ direct or generalized reciprocity,^74^ or by-product mutualism^2^).

## STAR★Methods

### Key resources table

**Table:** 

REAGENT or RESOURCE	SOURCE	IDENTIFIER
**Deposited data**		

Dataset	This study	Martínez, Mayte (2023), “Dogs Assurance Game data”, OSF data: https://doi.org/10.17605/OSF.IO/W5CJ9
R Code	This study	Martínez, Mayte (2023), “Dogs Assurance Game data”, OSF data: https://doi.org/10.17605/OSF.IO/W5CJ9

### Resource availability

#### Lead contact

Further information and any related request should be directed to and will be fulfilled by the lead contact, Mayte Martínez (mariateresa.martineznavarrete@vetmeduni.ac.at).

#### Materials availability

This study did not generate new unique reagents.

#### Data and code availability

Data used to fit models reported in this paper have been deposited on OSF and are publicly available as of the date of publication. DOI listed in the key resources table.

All original code has been deposited at OSF and is publicly available as of the date of publication. DOI listed in the key resources table.

Any additional information required to reanalyse the data reported in this paper is available from the lead contact upon request.

### Experimental models and subject details

#### Animals

We tested eleven dyads of pet dogs (13 females and 9 males, mean age ±SD: 5.19 ± 2.91 years) of varying breeds (see supplemental information, Table S1). Four additional dyads were unable to complete the training because one or both of the dogs lost interest in the task (3 dyads) or because the owner stopped participation (1 dyad). Dyads were formed by dogs living in the same household for at least 6 months that did not show aggression towards each other. Three further dogs participated as stooges in the training. The study was conducted at the Clever Dog Lab at the University of Veterinary Medicine Vienna in an empty test room (approx. 6 m × 2 m). All dogs participated in the different phases of the study (preference test, training, tolerance tests and Assurance game) in the same order (see supplemental information, Figures S2 and S3).

#### Ethics statement

This study was discussed and approved by the Ethik un Tierschutzkomission of the University of Veterinary Medicine Vienna (Approval number: 142/07/2019), and dog owners signed a consent form before participation.

### Method details

#### Food preference test

We tested the food preferences of each dog to establish which food was to be used as a high-value reward (HVR) and a low-value reward (LVR) in the Assurance game. The food preference test^54^^,^^75^ consisted of 12 trials in which the dogs could choose between two different foods (that they would eat) of different quality. If a dog chose the same food in at least 9 out of the 12 trials (binomial test, p < 0.02), we designated that food as the HVR food for the Assurance game and the non-preferred one as the LVR. We started all the food preference tests using a piece of dry food as the LVR versus an equal-size piece of sausage as HVR. If dogs failed to show a consistent preference, we tried again with a different combination of foods (for full details on the food preference test, see supplemental information, Figure S4). Because we tested the preferences of each dog individually, HVR and LVR were not necessarily the same for both members of the dyads. This resulted in six dyads in which both members have the same type of food as HVR and the same type of food as LVR; three dyads in which they had the same HVR but different LVR; and two dyads in which both members have different HVR and different LVR (see Table S1). We were unable to keep testing until we found foods for which the pair shared a preference because of dogs’ dietary restriction and time limitations.

#### Assurance game: Experimental set-up

In the Assurance game, there were two different sliding tables, one representing the *Hare* choice (*Hare* table) and the other representing the *Stag* choice (*Stag* table). The tables looked nearly identical from the dogs’ perspective (i.e., in both cases, their only option was to pull a rope, so their choice was which one to pull; see Figure 1). To facilitate the discrimination between the two apparatuses, we placed them on either a blue or a white carpet (counterbalanced between dyads). The *Hare* and the *Stag* tables were located across from one another, on opposite sides of the room (but each option was on the same side for both dogs, since the Stag table required joint action), and separated by a fence, so the dogs could see each other but could not access each other’s sides of the tables. Each dog was accompanied by either the owner or the experimenter (always the same for each dog), who sat equidistant between the *Hare* and the *Stag* tables (i.e., at the starting position), on their dog’s side of the fence, holding the dog on the collar at the start of each trial.

(a) *Hare* table. The *Hare* table was baited with LVR and could be solved individually. This apparatus consisted of two independent sliding tables (one for each dog) of approximately 50 × 50 cm. A Plexiglas cover ensured that the food was visible but could not be reached directly. To obtain it, dogs had to pull a rope attached to the sliding platform, which caused it to slide out, making the food accessible. Dogs could then access their food (independently of whether their partner chose *Hare* or *Stag*).
(b) *Stag* table. The *Stag* table was baited with HVR and could only be solved cooperatively. The apparatus consisted of one sliding table of approximately 100 × 50 cm that could be operated by simultaneously pulling the two ends of a rope that was threaded through metal rings attached to the sliding platform.^76^ If only one end of the rope was pulled, it caused the rope to slide out of the rings, making the rewards inaccessible. The apparatus was placed in the middle of the fence in a way that each dog could access only one end of the rope and half of the apparatus. Identical to the *Hare* table, a Plexiglas cover prevented the animals from accessing the rewards without releasing the sliding table.

#### Assurance game: Training

Each member of the dyad was separately trained to operate the *Hare* and the *Stag* tables (for full details of the training, see supplemental information, Figure S3). Food used in the training was always an LVR to prevent the dogs from developing a preference for one of the tables before the test. Each dog passed three training phases. In the *Hare* training, dogs learned to operate the *Hare* table. In the *Stag* training, dogs learned to solve the *Stag* table, first with the experimenter and then with a stooge dog acting as the partner. Additionally, to complete this training phase dogs had to be able to refrain from pulling their rope when their partner was delayed (up to 10 seconds). We included this phase to ensure that dogs knew that they could not solve the *Stag* table alone. Finally, in the *setup familiarization*, dogs were exposed to two trials of each apparatus (four trials total) baited with their corresponding food (HVR in the *Stag* table, LVR in the *Hare* table). In this way subjects learned the contingencies in forced choice trials so that they experienced each one the same number of times.

At the end of the training, we assigned the role of Subject 1 (S1) or Subject 2 (S2) to each member of the dyad. In the Assurance game test trials, S2 was released one second after S1. Therefore, S2 always had the opportunity to see where S1 was heading before making its own choice. We always assigned the role of S2 to dogs that completed all the steps in the training, including the training with the stooge dog. For the dyads in which both individuals met that criterion, the roles of Subjects 1 and 2 were randomly assigned.

#### Assurance game: Test sessions

Dogs completed two sessions of the Assurance game, each consisting of 4 forced-choice warm-up trials and 40 test trials, with a break after 20 trials. During those breaks, dogs were free to move in the room and interact with each other and with the owner. To account for the effect of side bias on the dogs’ performance, we ran the second session on a different day, identical to the first session with the exception that the position of the tables was reversed (i.e., if the *Stag* table was on the left in the first session, we placed it on the right side of the room for the second session).

##### Warm-up trials

These trials were forced choice trials conducted to ensure the dogs were aware of the position of the *Stag* and *Hare* tables. In these trials, dogs could not choose between *Stag* or *Hare*, as only one of the tables (alternating *Stag* and *Hare* table) was baited with food while the other was both empty and made inoperative by coiling the rope away from the dogs. Which table was baited in the first warm-up trial was counterbalanced across dyads. In the warm-up trials the dogs could freely move within their side of the room while the experimenter put the corresponding food (HVR for the *Stag* table, LVR for the *Hare* table) on the apparatus and stepped away. At this point, the dogs were allowed to retrieve it (individually in the case of the *Hare* table and pulling together in the *Stag* table).

##### Test trials

All trials began with S2 being held by the experimenter and S1 by the owner at the starting position. The experimenter and the owner were instructed to look straight at each other, to avoid unintentionally influencing the dogs’ choice by gazing toward one of the tables. When the experimenter said “*Ok*”, the owner released S1 and, one second after, the experimenter released S2. A choice was defined as the animal pulling one of the ropes (e.g., if a dog pulled the rope of the *Hare* table, that was considered a *Hare* choice). Once dogs started pulling one of the ropes, they were allowed to pull until they obtained the rewards (if a dog chose *Hare* or both dogs chose *Stag)*, or until they pulled the entire length of the rope out and the table could no longer be solved (if a dog chose *Stag* but their partner had chosen *Hare*). Dogs were not allowed to make a second choice. If, after pulling the rope in one table, the dog attempted to reach the other table, the experimenter approached that table and wound the rope inside the apparatus, out of the dog's reach. The trial ended once the two dogs had made a choice or after 40 seconds, at which point the owner and experimenter called the dogs back to the starting position. Then, the experimenter left S2 in the starting position and went to re-bait the apparatuses. The experimenter visited all the tables independently of whether they were empty or still baited after the previous trial, starting with a different table in each trial. After that, the experimenter returned to the starting position and a new trial began; this was repeated until all 40 trials were completed.

#### Tolerance test

To evaluate if coordination in the Assurance game affected the dogs' attitude towards their partners, we assessed whether the dyad's tolerance levels in a food context, measured as time feeding together on a food resource, changed after the Assurance game. For this reason, we conducted one tolerance test after each of the Assurance game sessions to determine whether dogs that coordinated more on *Stag* (measured as the amount of HVR that they ate in the Assurance game), were more willing to co-feed during the tolerance test. Even if that prediction was confirmed, that does not necessarily mean that dogs are changing their attitudes towards their partners because they understood their role in obtaining the HVR during the Assurance game. Instead, they may co-feed more merely because they are more satiated by the HVR eaten during the Assurance game or because they developed a positive association between the presence of the partner and the HVR. Those last two scenarios do not involve any understanding of the game. To account for this, we included three additional tolerance tests, conducted in three different days immediately after the training sessions. To control for the effect of the HVR that they receive during the experimental sessions, in these subsequent three sessions dogs were fed variable amounts of HVR (none, 20 or 40, order counterbalanced between dyads) before the tolerance tests (once the training was over, to avoid interfering with it). If dogs changed their attitudes towards their partners after successful coordination in the Assurance game, dogs co-feeding would be influenced by the amount of HVR eaten before the test in the Assurance game, but not by the amount of HVR eaten before the test in the training days.

In the tolerance tests,^54^ dogs were presented with a single bowl filled with one cup of dry food. The bowl was covered with a cardboard box (see supplemental information, Figure S5), attached to a rope threaded through a hook in the ceiling, in a way that the experimenter could lift the box from a distance (1.5 m from the box), to avoid the potential interference of a human in close proximity. The experimenter lifted the box when both animals were within 10 cm of the box, allowing them to approach and eat the food. The test ended when there was no food left or when both dogs were more than one body length away from the bowl.

### Quantification and statistical analysis

All tests were video-recorded for later coding. For each individual in every trial of the Assurance game, we coded the choice (*Hare*, *Stag,* or *no choice*), and whether S1 or S2 was the first one to move two steps towards the *Stag* or *Hare* table (see Data S1). A second person, blind to the aim of the study, coded 20% of the videos, revealing excellent consistency between coders (Intra-class correlation coefficient for co-feeding time = 0.953, Cohen’s kappa for dog's choice and which subject moves first = 1).

Statistical analyses were done after excluding the trials in which one or both members of the dyad did not make a choice. Average proportion of missing trials per dyad was 0.04 ± 0.11. We tested whether dyads’ choices of each of the four possible outcomes (*Stag-Stag*, *Hare-Hare*, *Hare-Stag*, and *Stag-Hare*) in each session were different from chance. To do this we used chi-square goodness-of-fit and, as there were four possible outcomes, we set the chance level at 25%. Whenever the chi-square was significant (p < 0.05), we checked the standardized residuals to assess which of the four outcomes differed from chance. Standardized residuals of +/− 2.58 were considered statistically significant (p < 0.01).^55^

We explored which factors influenced whether dyads found the payoff-dominant NE (*Stag-Stag*) with a binomial Generalized Linear Mixed Model (GLMM),^77^ the *Stag-Stag model*. Whether the dyad's choice was *Stag-Stag* in each trial was the response variable. To test if more tolerant dyads were more likely to choose *Stag-Stag*, we included the proportion of co-feeding in the tolerance tests before the Assurance game (one single measure derived from averaging their proportion of co-feeding across the first three tolerance tests). To test for learning effects, we included trial and session numbers as predictors in the model. Finally, to test for the effect of age, we included the S1 and S2’s age as predictors. We also added the three-way interactions between trial number, session number, and the rest of the variables (i.e., trial x session x tolerance, trial x session x S1’s age, trial x session x S2’s age).

To assess whether the subjects adjusted their behaviour to each other, we ran two different binomial GLMMs. In the first one (*S2 model*), we tested whether S2’s (the second dog to be released) choice of *Stag* or *Hare* was influenced by S1’s (the first dog to be released) choice. In this model the choice of S2 was the response variable and S1’s choice was used as predictor. We also included the fixed effects of trial, and whether S2 received a reward in the previous trial (to control for the possibility of dogs maintaining/changing their choices solely depending on whether they were previously rewarded for that choice). Additionally, as certain dyads consistently coordinated on *Stag* in both sessions, we were concerned that this lack of variability could influence the results. Thus, add the variable “dyad type” to the model. This variable was used to define two types of dyads: 1) dyads that coordinated on *Stag* in both sessions, and 2) other dyads (including side biased dyads and one dyad that coordinated on *Stag* in the first session and made uncoordinated choices in the second session). We also included all the two-way interactions between the predictor (S1’s choice), and the control variables (rest of the variables in the model).

To explore whether S1 was adjusting their choices to S2’s, we ran a GLMM testing whether S1’s choice (response variable) was influenced by S2’s choice in the previous trial (predictor). We used the S2’s choice in the previous trial instead of the current trial because S1 was always the first one to choose (in our data, S1 never delayed, so S2 was always the first one to choose). However, S1’s choice could take into account S2’s choice in the previous trial (e.g., if in trial *n-1*, S2 chose *Hare*, this may encourage S1 to also choose *Hare* in trial *n*). We also included the fixed effects of trial number and dyad type, together with the two-way interactions between these variables and the predictor. In the *S1 model* we could not include whether S1 was rewarded in the previous trial because that variable was redundant with Subject's 2 choice in the previous trial (i.e., most of the trials in which S2 chose *Stag* resulted in a reward for S1 because they led to successful coordination).

Because S2 was released after S1, our models assumed that S2 could always see where S1 was heading before making its own choice. However, we notice that in a few trials S1 was not always the first one to move. Thus, we removed the 50 trials that did not meet that assumption from our sample before fitting the *S1 and S2 models* (in 8 trials that information was not available because of camera malfunction, in 4 trials both dogs moved virtually at the same time, and in 38 trials S2 started moving first).

Finally, we explored whether successful coordination in the payoff-dominant NE influenced the subject’s behaviour towards their partner after the game. In the *Tolerance model* we used the proportion of time that dog dyads spent feeding together in each tolerance test as a response variable. For this model we used a zero-inflated beta GLMM (45.5% of the tolerance tests resulted in no co-feeding). We aimed to test whether the amount of high value reward (HVR) eaten before the tolerance test led to more tolerance (co-feeding) among the partners, but only if this HVR was obtained by coordinating in the Assurance game, in contrast with the days in which the HVR was given by the experimenter before the tolerance test (i.e., training days). For this reason, we included both the number of HVR eaten before the tolerance test and condition (whether it was a training or a testing day) in the model. We aimed to test for the significance of the interaction between these two variables.

All the GLMMs included dyad identity as a random intercept, and all the identifiable random slopes within dyad (see supplemental information and Table S2 for a detailed description of the model’s construction). Additionally, to account for the variability in dyads sex composition, we included in all the models a random factor with S1 and S2 sex (i.e., four levels: malemale, malefemale, femalefemale, femalemale). We used a full-null model comparison approach, comparing the models through likelihood ratio tests^77^ with models lacking the predictors but otherwise identical.

Statistical analyses were conducted in R 4.0.5.^78^ For the GLMMs we used the package “lme4”^79^ whenever the response variable was dichotomous, and “glmmTMB”^80^ when it was a proportion.

## Supplemental information

Document S1. Figures S1–S10 and Tables S1–S7

Data S1. Data used for analyses, related to STAR Methods
