# Supplementary material for: Some dogs can find the payoff-dominant outcome in the Assurance game
Source: iScience. 2023 Dec 9;27(1):108698. doi: 10.1016/j.isci.2023.108698 (PMC10776926; doi:10.1016/j.isci.2023.108698)
Supplement: Document S1. Figures S1–S10 and Tables S1–S7 [file mmc1.pdf]

iScience, Volume *27*

Supplemental information

Some dogs can find the payoff-dominant outcome in the Assurance game

Mayte Martínez, Selina Schöndorfer, Lauren M. Robinson, Sarah F. Brosnan, and Friederike Range

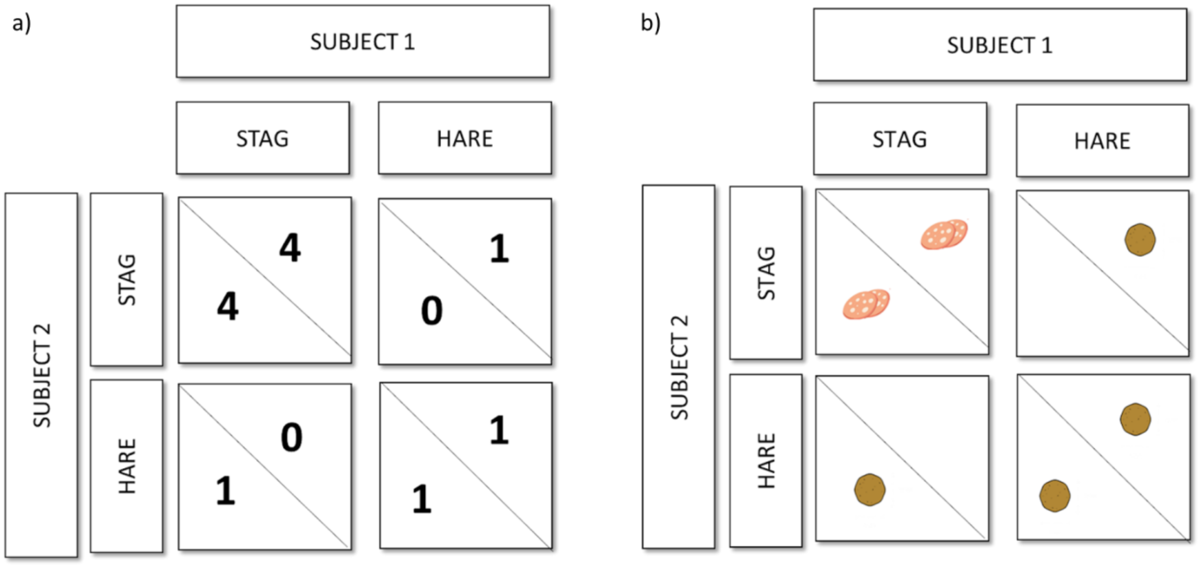

Figure S1. Assurance game's payoff matrices, related to STAR Methods. Payoff matrix typically used in the Assurance game with non-human primates (a), and payoff matrix used in the current study (b).

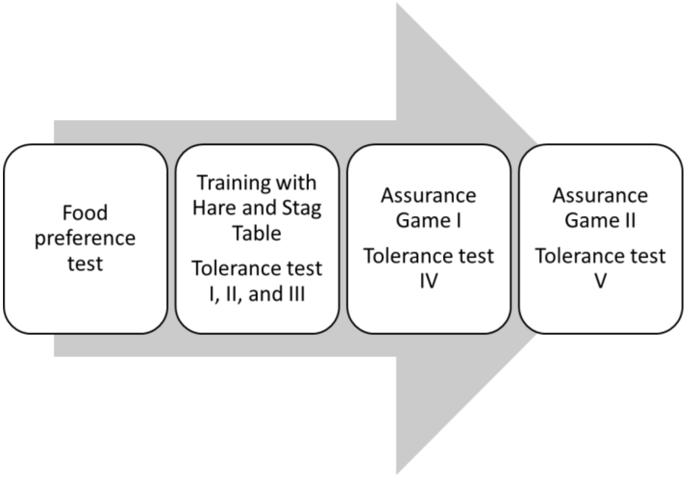

Figure S2. Schematic depiction of the testing schedule, related to STAR Methods. See also Figure 3.

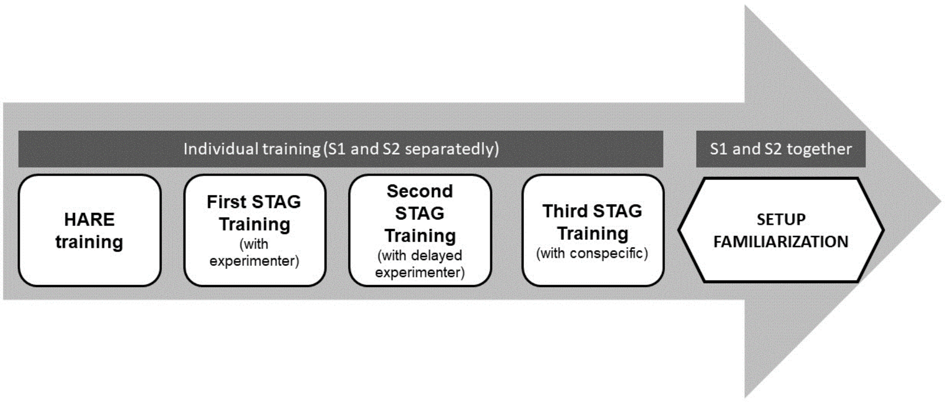

Figure S3. Schematic depiction of the training schedule, related to STAR Methods. See also Figure 2. Each member of the dyad was separately trained to operate the *Hare* and the *Stag* tables using shaping and positive reinforcement. During the training, both tables were in the room, but only the apparatus with which the training was being performed was baited with food. The remaining table(s) were made inaccessible by coiling the rope out of reach.

Hare training: Dogs were first trained to pull the rope that solved the *Hare* table until they were able to successfully retrieve the food from the apparatus five consecutive times without any help from the experimenter. On average, dogs reached this criterion within 2.55 training sessions of 20 minutes (*SD* = 1.74, range 1 to 8 sessions).

First Stag training: Afterwards, we trained the dogs to operate the *Stag* table with the experimenter as a partner. For the first *Stag* training, the experimenter held one end of the rope and waited for the dog to approach and pull the other end to make the rewards accessible. If the dog did not approach, the experimenter called the dog and gave it the command to pull.

Second Stag training: Once dogs succeeded to pull the rope at the same time as the experimenter without any command in two consecutive trials, we verified that they understood that two individuals were required to solve the apparatus with the second *Stag* training. To do this, we ensured that they would wait for their partner to arrive at the apparatus instead of pulling the rope alone (which would cause the food to be inaccessible for both individuals). For this, the experimenter did not hold the rope from the beginning of the trial but she stayed one step away from the apparatus and only approached and held the rope after five seconds. When dogs were able to wait and then approach and pull at the same time as the experimenter in three consecutive trials, we increased the delay to ten seconds and repeated the same procedure. It took an average of 65.22 trials (*SD*=29.48), distributed across 1 to 4 20-min sessions (*average*=2.18, *SD*=0.67) for the dogs to meet criterion on the training with the experimenter partner.

Third Stag training: For the third *Stag* training, we repeated the training on the *Stag* table with a conspecific stooge that had been previously trained to pull the rope reliably. In these trials, the subject was released and the experimenter held the stooge back for 5 seconds and, if they succeeded in this, 10 seconds. Out of our 22 dogs, 18 were able to successfully complete this training phase. These dogs finished the training with the conspecific stooge after an average of 31.71 trials (*SD*=23.11), distributed across one to three sessions (*average*=1.67, *SD*=0.74). From our final sample of 22 dogs, four individuals did not complete this training stage because they showed aggressive behavior (growling) towards the stooge. We switched these four individuals so that they performed the last training stage with the experimenter as a partner while the rest of the dogs completed it with the conspecific stooge (see supplemental material, table S1).

Setup familiarization: Finally, in an additional training stage, dogs were exposed to both apparatuses baited with their corresponding food (HVR in the *Stag* table, LVR in the *Hare* table, only one of the two options was baited in each trial). This training stage consisted of four trials, two consecutive trials on the *Stag* table and two consecutive trials on the *Hare* table (order counterbalanced between individuals). The participant dog and the stooge were released at the same time and allowed to obtain the food from either the *Stag* or the *Hare* table, depending on the trial, while the other table remained empty and unsolvable (the rope was coiled).

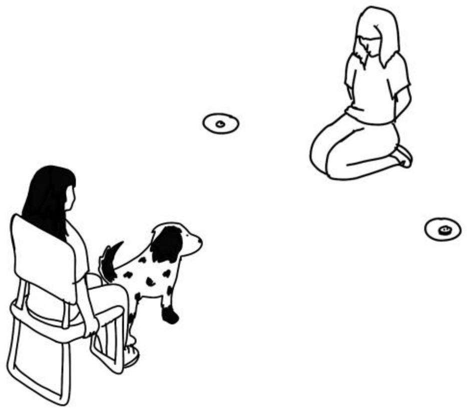

Figure S4. Setup for the preference test, related to STAR Methods. We tested the food preferences of each dog to establish which food was to be used as a high-value reward (HVR) and a low-value reward (LVR) in the Assurance game. Because we wanted the dogs to consider both types of food as a reward, we first allowed the dogs to try them, to confirm they would eat them. The food preference test consisted of 12 trials in which the dogs could choose between two different foods (that they would eat) of different quality. Each trial started with the dog kept on a leash next to the owner, who sat on a chair in front of them (1m away from the dog). The experimenter put the two different foods in two plastic lids and showed them to the dog, allowing them to sniff the lids. She then placed them on the ground, 50cm apart from each other. As soon as the experimenter left the food on the ground, she looked down to avoid influencing the dog. This was the signal for the owner to release the dog, who was then free to approach and eat one of the two foods. As soon as the dog touched one of the foods, the experimenter removed the other one. The side on which the experimenter showed each food was alternated in each trial to control for side bias. If a dog chose the same food in at least 9 out of the 12 trials (binomial test, $p<0.02$), we designated that food as the HVR food for the Assurance game and the non-preferred one as the LVR.

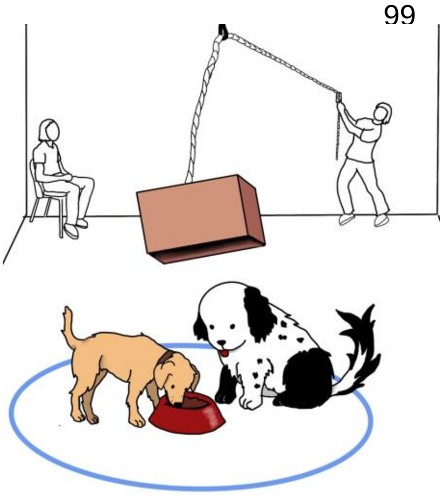

Figure S5. Setup for the tolerance test, related to STAR Methods. Dogs had simultaneous access to a bowl with food until food was over or until both dogs were more than one body length away from the bowl. We initially coded the occurrence of any aggressive behavior (displacements and growling), but we decided to exclude that measure from the analysis because those behaviors hardly ever occurred (across all tests and dyads, we found only one growl in one dyad and three displacements in another dyad). We also coded whether and for how long each dog was feeding alone, but we did not use that information because it was redundant with the dogs hierarchy (dominant dogs tend to feed alone at the start of the session) and did not add information related to our hypothesis (more coordination = more co-feeding).

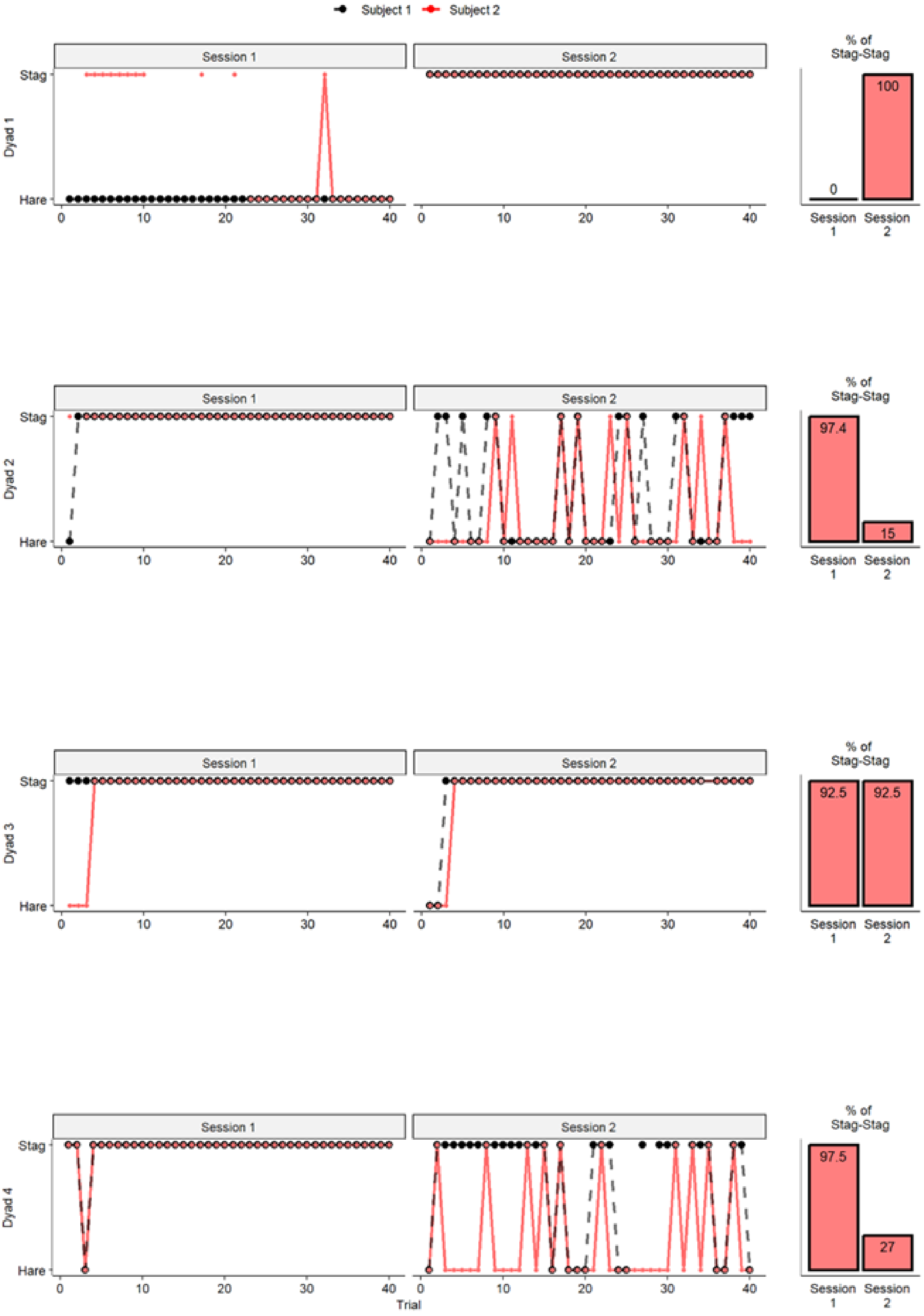

Figure S6. Individual choices by trial in the Assurance Game for dyads 1 to 4, related to STAR Methods. See also Figures S5 and S6. Each dot represents the S1's (black) or S2's (red) choice in each trial. Blank spaces indicate trials in which the individual did not make any choice. The bar plot on the right shows the percentage of Stag-Stag choices (calculated excluding trials in which either S1 or S2 did not make a choice) by session.

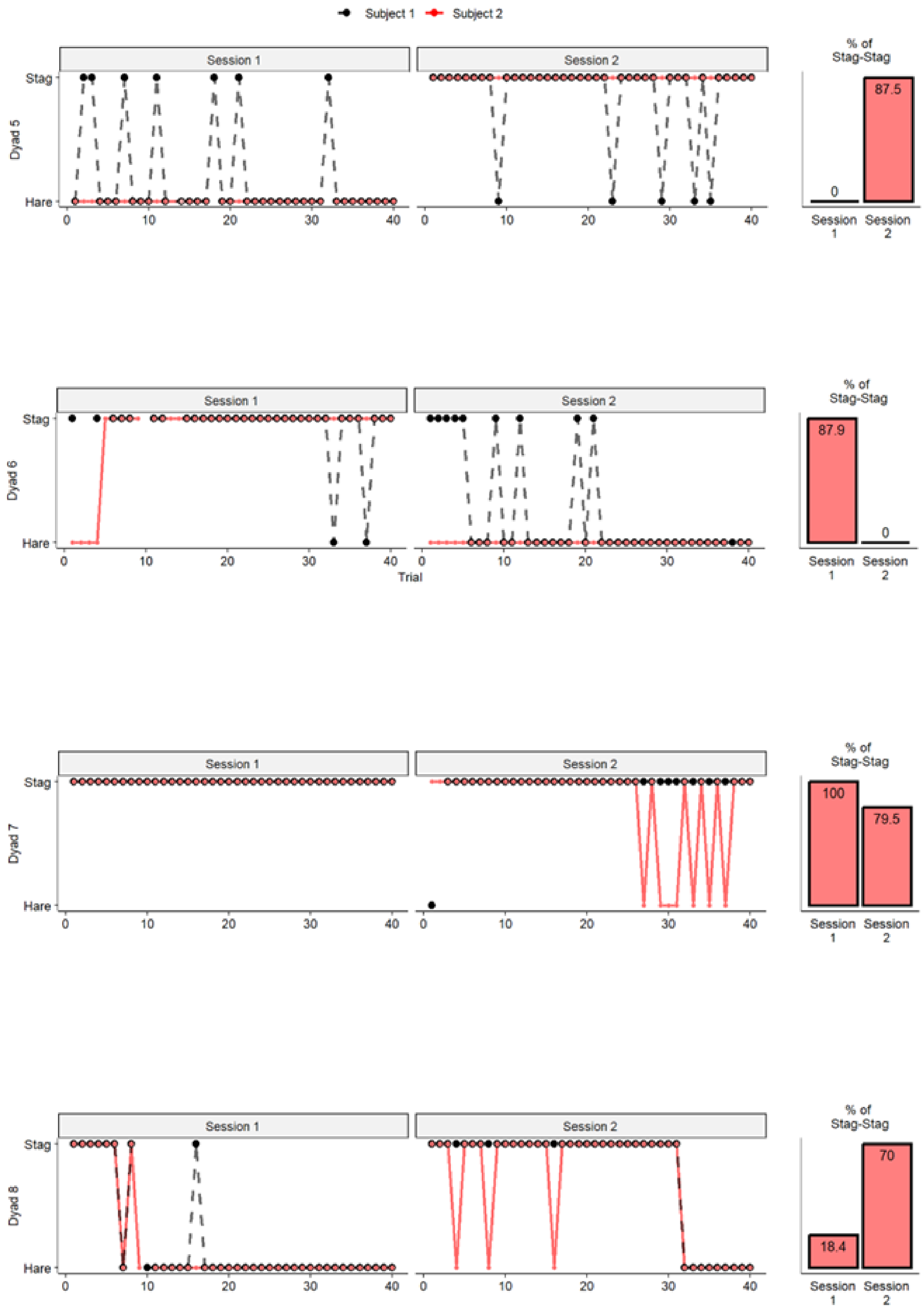

Figure S7. Individual choices by trial in the Assurance Game for dyads 5 to 8, related to STAR Methods. See also Figures S4 and S6. Each dot represents the S1's (black) or S2's (red) choice in each trial. Blank spaces indicate trials in which the individual did not make any choice. The bar plot on the right shows the percentage of Stag-Stag choices (calculated excluding trials in which either S1 or S2 did not make a choice) by session.

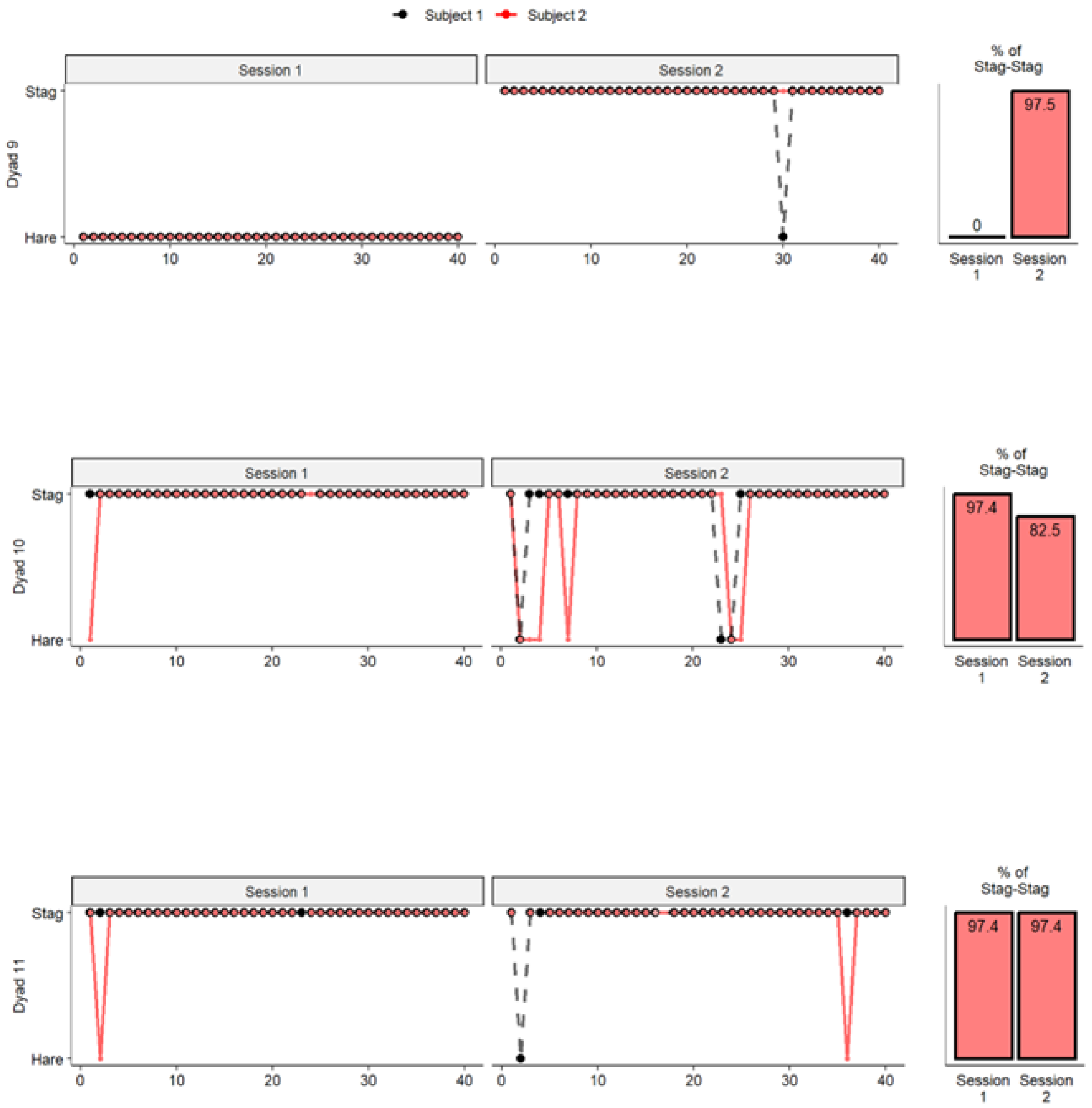

Figure S8. Individual choices by trial in the Assurance Game for dyads 9 to 11, related to STAR Methods. See also Figures S4 and S5. Each dot represents the S1's (black) or S2's (red) choice in each trial. Blank spaces indicate trials in which the individual did not make any choice. The bar plot on the right shows the percentage of Stag-Stag choices (calculated excluding trials in which either S1 or S2 did not make a choice) by session.

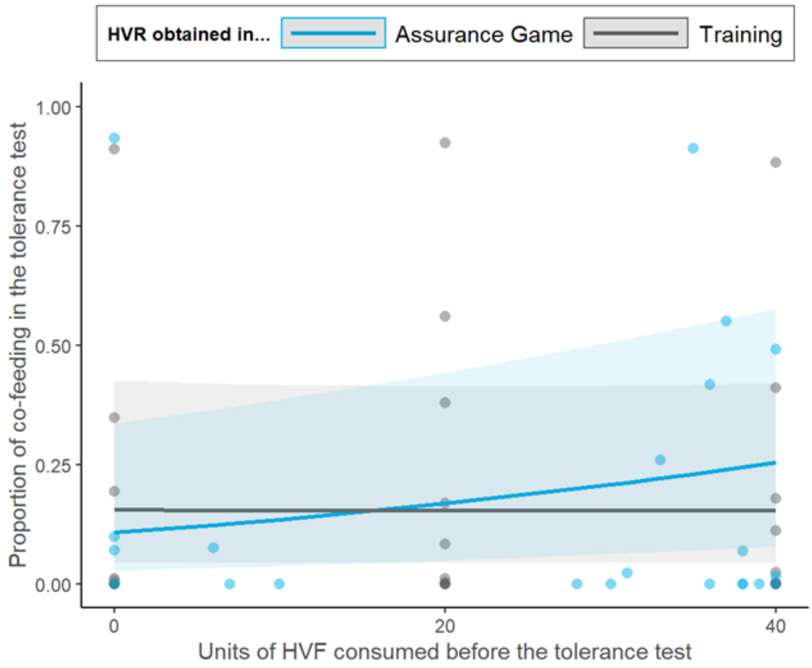

Figure S9. Proportion of co-feeding depending on condition (Assurance Game or training) and amount of high value food consumed before the test, related to STAR Methods. Shadowed area corresponds to 95% Wald confidence intervals.

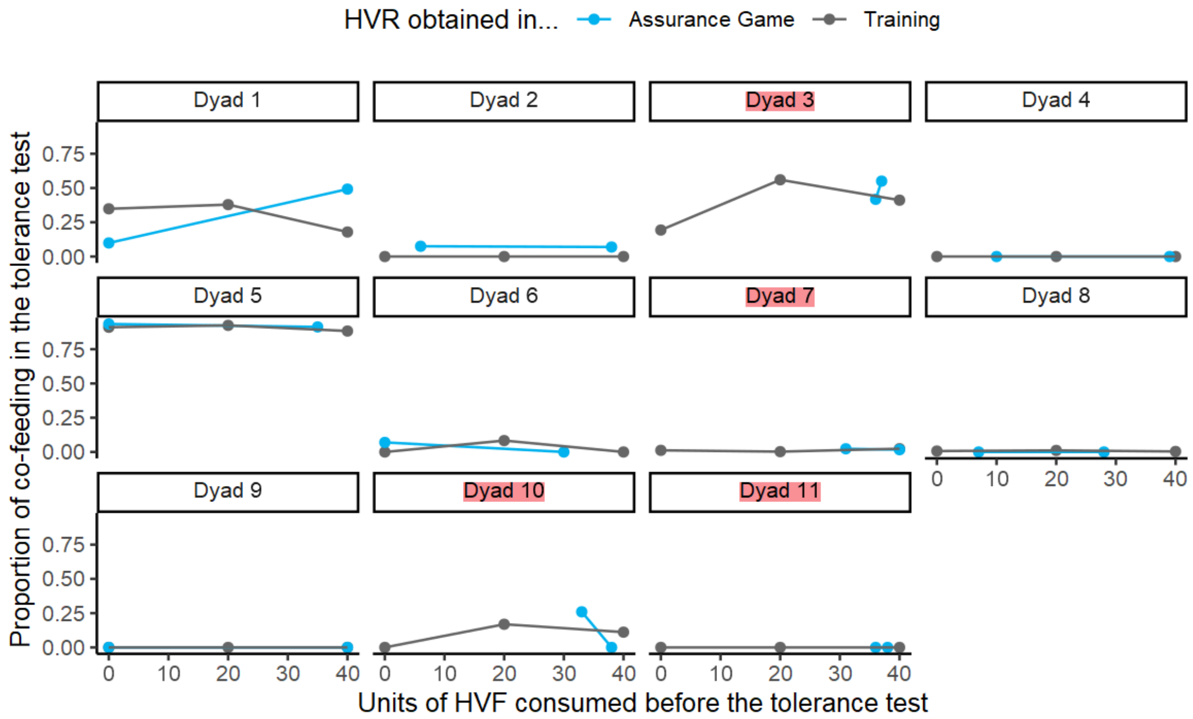

Figure S10. Co-feeding by dyad depending on condition (Assurance Game or training) and amount of high value food consumed before the test, related to STAR Methods. The names of the dyads that consistently coordinated on *Stag-Stag* are highlighted.

Table S1. List of participants in the study, including information regarding dyad number, sex, breed, age, reward used and stooge. Related to STAR Methods.

Dyad	Name	Rol[1]	Age (years)	Sex[2]	Breed	LVR	HVR[3]	Stooge dog
1	Prim	S1	5	F	Shetland sheepdog	Dry food	Sausage (2)	Leia 2
	**Annie**[4]	S2	8.17	F	Shetland sheepdog	Dry food	Sausage (2)	Leia 2
2	Dawin	S1	5.08	M	Border collie	Carrot	Sausage	Leia 2
	**Benny**	S2	7.67	M	Border collie	Dry food	Sausage (2)	Leia 2
3	**Alou**	S1	4.92	F	Australian shepherd	Lettuce	Sausage	NA
	Crash	S2	2.92	M	Australian shepherd	Zucchini	Cheese (2)	Leia 2
4	**Lenny**	S1	6.58	M	Canarian warren hound	Zucchini	Sausage	Leia 2
	Gandalf	S2	1.58	M	Andalusian hound	Dry food	Sausage	Ultimo
5	**Dora**	S1	9.17	F	Hovawart	Dry food	Sausage (2)	Leia 2
	Helsa	S2	2.17	F	Hovawart	Dry food	Sausage	Leia 2
6	Joseph	S1	3.33	M	Mixed breed	Dry food	Sausage	Leia 2
	**Josephine**	S2	4.17	F	Pumi	Dry food	Sausage	Leia 2
7	**Eowyn**	S1	3.50	F	Mixed breed	Dry food	Sausage	NA
	Leia	S2	9.50	F	Beagle	Dry food	Sausage (2)	Leia 2
8	**Hetti**	S1	2.17	F	Mixed breed	Dry food	Sausage	NA
	Matteo	S2	1.67	M	Segugio italiano	Dry food	Sausage	Leia 2
9	**Cheynna**	S1	7.17	F	Australian shepherd	Dry food	Sausage	NA
	Mavie	S2	1.92	F	Border collie	Sausage	Dry food	Leia 2
10	Tiara	S1	7.67	F	Border collie	Zucchini	Sausage (2)	Vega

	**Miley**	S2	11.58	F	Border collie	Dry food	Sausage (2)	Vega
11	Mailo	S1	5.92	M	Mixed breed	Dry food	Sausage	Leia 2
	**Timo**	S2	2.33	M	Mixed breed	Dry food	Sausage	Leia 2

[1]S1=Subject 1. S2= S2.
[2] F=Female. M=Male.
[3]Only one piece of food was used, unless a different number is indicated in brackets.
[4] Dominant individuals in each dyad (reported by owner) are indicated in bold.

Table S2. Description and details of the GLMMs, related to STAR Methods.

	***Stag-Stag* model**	**S2 model**	**S1 model**	**Tolerance model**
**Response (error structure)** [1]	Dyad choice of *Stag-Stag*: y/n (binomial)	S2`s choice: *Stag/Hare* (binomial)	S1`s choice: *Stag/Hare* (binomial)	Co-feeding: proportion (zero inflated beta)
**Fixed effects (full model)**	session * trial * (co-feeding +s1's age + s2's age)	S1's choice * (session + trial + reward in previous trial)	subject 2's choice in the previous trial * (session + trial)	number of pieces of HVR * condition(test/training)
**Random effects**	(1 + session * trial || dyad ID) + (1|dyad_sex)	(1 + S1's choice * (session + trial + reward in previous trial) || dyad ID)	(1 + Subject 2's choice in the previous trial * (session + trial) || dyad ID)	(1 +number of pieces of HVR + condition || dyad ID)

[1]After we fit all the models, we followed the same procedure with all of them. First, we used a full-null model comparison approach to avoid false positives derived from multiple testing [S1], comparing the models through likelihood ratio tests [S2] with models lacking the predictors but otherwise identical. P-values were estimated via single deletion (LRT method). Model stability was estimated by dropping the individuals one at a time from the data and comparing the estimates derived from models fitted to these subsets with those in the full model. The *S2* and *S1 models* were fairly stable, with the exception of the estimates for dyad type and its interactions with other variables. The *Stag-Stag* was unstable, especially regarding the effect of co-feeding and its interactions with other variables. The *Tolerance model* was moderately unstable, which may indicate the existence of influential cases. When we inspected the Variance Inflation Factors (VIF; [S3]) using the package "car" [S4], we did not find any problem derived from collinearity among the predictors (all VIF were below 1.5). Confidence intervals were obtained using the Wald intervals.

Table S3. Dog pairs' responses in the Assurance Game, related to STAR Methods.

**Dyad number**	**Session**	**Dyad' s choices[1]**				**χ^2**	***p-value***	***Missing trials[3]***
		Stag-Stag	***Hare-Hare***	***Stag-Hare***	***Hare-Stag***			
1	1	0 (*-2.646*)	17 (*3.780*)	0 (*-2.646*)	11 (*1.512*)	30.571	<0.001	S1:Hare+ S2:None (12)
	2	40 (*9.487*)	0 (*-3.162*)	0 (*-3.162*)	0 (*-3.162*)	120.000	<0.001	NA
2	1	38 (*9.047*)	0 (*-3.122*)	0 (*-3.122*)	1 (*-2.802*)	109.205	<0.001	S1:Stag S2:None (1)
	2	6 (*-1.265*)	21 (*3.479*)	10 (*0.000*)	3 (*-2.214*)	18.600	<0.001	NA
3	1	37 (*8.538*)	0 (*-3.162*)	3 (*-2.214*)	0 (*-3.162*)	97.800	<0.001	NA
	2	37 (*8.538*)	2 (*-2.530*)	1 (*-2.846*)	0 (*-3.162*)	97.400	<0.001	NA
4	1	39 (*9.171*)	1 (*-2.846*)	0 (*-3.162*)	0 (*-3.162*)	112.200	<0.001	NA
	2	10 (*0.247*)	10 (*0.247*)	17 (*2.548*)	0 (-3.041)	15.865	0.001	S1:None +S1:Hare (3)
5	1	0 (*-3.082*)	33 (*7.624*)	5 (*-1.460*)	0 (*-3.082*)	79.263	<0.001	S1:Stag + S2:None (2)
	2	35 (*7.906*)	0 (*-3.162*)	0 (*-3.162*)	5 (*-1.581*)	85.000	<0.001	NA
6	1	29 (*7.224*)	0 (*-2.872*)	2 (*-2.176*)	2 (*-2.176*)	69.909	<0.001	S1:None + S2:Hare (2) / S1:None + S2:None (1) / S1:None + S2:Stag (4)
	2	0 (*-3.122*)	30 (*6.485*)	9 (*-0.240*)	0 (*-3.122*)	61.615	<0.001	S1:Hare+S2: None (1)
7	1	40 (*9.487*)	0 (*-3.162*)	0 (*-3.162*)	0 (*-3.162*)	120.000	<0.001	NA
	2	31 (*6.805*)	0 (*-3.122*)	7 (*-0.881*)	1 (*-2.802*)	64.692	<0.001	S1:None+S2: Stag (1)
8	1	7 (*-0.811*)	30 (*6.651*)	1 (*-2.758*)	0 (*-3.082*)	62.000	<0.001	S1:Hare + S2:None (1) / S1:None + S2:Hare (1)

	2	28 (*5.692*)	9 (*-0.316*)	3 (*-2.214*)	0 (*-3.162*)	47.400	<0.001	NA
9	1	0 (*-3.162*)	40 (*9.487*)	0 (*-3.162*)	0 (*-3.162*)	120.000	<0.001	NA
	2	39 (*9.171*)	0 (*-3.162*)	0 (*-3.162*)	1 (*-2.846*)	112.200	<0.001	NA
10	1	38 (*9.047*)	0 (*-3.122*)	1 (*-2.802*)	0 (*-3.122*)	109.205	<0.001	S1:None + S2:Stag (1)
	2	33 (*7.273*)	2 (*-2.530*)	4 (*-1.897*)	1 (*-2.846*)	71.000	<0.001	NA
11	1	38 (*9.047*)	0 (*-3.122*)	1 (*-2.802*)	0 (*-3.122*)	109.205	<0.001	S1:Stag + S2:None (1)
	2	37 (*8.922*)	0 (*-3.082*)	1 (*-2.758*)	0 (*-3.082*)	106.211	<0.001	S1:Hare + S2:None (1) / S1:Stag + S2:None (1)

[1] Numbers outside the brackets indicate the number of trials in which dyads chose each outcome. Standardized residuals appear in italics within brackets, indicating whether that proportion of choices within the session deviates from chance (+/-2.58 = p<0.001).

[2] Number of trials between brackets. These missing trials (trials in which either S1, S2, or both did not make a choice) were removed from the sample before calculating the $\chi 2$.

Table S4. Results of the *Stag-Stag* model, related to STAR Methods.

Term	Estimate	SE	CI		Model stability		χ^2	df	*p*[1]
			Lower	Upper	Min	Max			
Intercept	0.47	1.22	-1.92	2.87	-81.75	16.06			
Co-feeding[2]	-2.75	1.42	-5.54	0.04	-175.39	19.60	1.36	1	0.244
Session(2)[3]	1.02	1.94	-2.78	4.82	-9.19	82.86	0.28	1	0.594
Trial[2]	0.78	0.53	-0.27	1.82	-47.57	11.06	1.30	1	0.255
S1's age[2]	0.55	1.23	-1.86	2.96	-6.00	2.85	0.80	1	0.372
S2's age[2]	1.50	1.25	-0.95	3.95	0.66	22.50	1.91	1	0.167
Session(2)*Trial	-0.65	0.53	-1.70	0.39	-4.92	47.40	1.53	1	0.216
Co-feeding*Session(2)	3.69	2.12	-0.45	7.84	-4.75	175.93	2.81	1	0.094
Session(2)*S1's age	0.21	1.98	-0.40	1.41	-1.81	6.63	0.01	1	0.915
Session(2)*S2's age	-1.00	1.96	-0.47	1.59	-22.05	0.29	0.25	1	0.615
Co-feeding*Trial	0.17	0.72	-1.25	1.58	-102.37	14.42	0.04	1	0.835
Trial*S1's age	0.50	0.46	-0.40	1.41	-0.84	1.94	0.91	1	0.341
Trial*S2's age	0.56	0.52	-0.47	1.59	-0.19	13.12	0.97	1	0.324
Co-feeding*Session* Trial	-0.15	0.71	-1.55	1.25	-4.09	102.11	0.04	1	0.835
S1's age *Session* Trial	-0.26	0.45	-1.14	0.61	-1.25	1.42	0.36	1	0.551
S2's age *Session* Trial	-0.33	0.49	-1.29	0.62	-12.78	0.29	0.46	1	0.495

[1]Some p-values are not indicated due to their limited interpretation.

[2]Continuous fixed effects were z-transformed to a mean zero and a standard deviation of one; original means(SD) were trial: 20.50(11.55), S1's age: 6.02(2.64), S2's age: 4.88(3.48), and co-feeding: 0.16(0.27)

[3] Reference level for Session is Session 1

Table S5. Results of the S2 model, related to STAR Methods.

Term	Estimate	SE	CI		Model stability		χ^2	df	*p*[1]
			**Lower**	**Upper**	**Min**	**Max**			
Intercept	-3.44	0.67	-4.74	-2.14	-4.30	-3.04			
S1's choice (*Stag*)[2]	5.97	0.77	4.44	7.46	5.09	7.45	18.34	1	**<0.001**
Dyad type (coordinates)[2]	2.37	1.77	-1.09	5.83	-11.71	4.58	3.15	1	0.076
Trial[3]	0.35	0.53	-0.68	1.37	0.12	0.81	1.59	1	0.208
Reward in previous trial (no)[2]	0.94	0.89	-0.79	2.68	-0.41	1.37	0.55	1	0.458
S1's choice * Dyad type	-1.25	1.80	-4.77	2.28	-3.71	14.21	0.47	1	0.492
S1's choice * Trial	0.35	0.45	-0.52	1.22	0.04	0.55	0.60	1	0.439
S1's choice * reward in previous trial	-0.78	1.11	-2.95	1.40	-1.306	1.39	0.32	1	0.575

[1] Some p-values are not indicated due to their limited interpretation.

[2] Reference level for S1's choice is *Hare*, for dyad type is Other (i.e. dyad did not coordinate in both sessions), and for reward in the previous trial is Yes.

[3] Continuous fixed effects were z-transformed to a mean zero and a standard deviation of one; original means(SD) was trial: 20.70(11.55).

Table S6. Results of the S1 model, related to STAR Methods.

Term	Estimate	SE	CI		Model stability		χ^2	df	*p*[1]
			Lower	Upper	Min	Max			
Intercept	-2.05	0.88	-3.78	-0.33	-3.45	-0.69			
Previous Subject 2's choice (*Stag*) [2]	5.10	1.24	2.66	7.53	3.16	6.86	4.01	1	**0.045**
Dyad type (coordinates)[2]	6.22	2.13	2.06	10.39	2.59	20.97	22.39	1	**<0.001**
Trial[3]	-0.96	0.40	-1.75	-0.18	-1.67	-0.70	2.80	1	0.94
Previous Subject 2's choice* Dyad type	-4.51	2.50	-9.40	0.39	-19.63	0.31	3.51	1	0.061
Previous Subject 2 choice* Trial	0.75	0.41	-0.05	1.55	0.55	1.16	3.58	1	0.058

[1] Some p-values are not indicated due to their limited interpretation.

[2] Reference level for Previous S1's choice is *Hare*, and for dyad type is Other (i.e. dyad did not coordinate in both sessions).

[3] Continuous fixed effects were z-transformed to a mean zero and a standard deviation of one; original means(SD) was trial: 21.24(11.25).

Table S7. Results of the Tolerance model, related to STAR Methods.

Term	Estimate	SE	CI		Model stability		χ2	df	*p*[1]
			Lower	Upper	Min	Max			
Intercept	-1.54	0.69	-2.90	-0.18	-2.16	-1.20			
HVR eaten[2]	0.43	0.15	0.13	0.73	0.08	0.58			
Condition (training)[2]	-0.16	0.20	-0.55	0.22	-0.36	-0.05			
HVR eaten * Condition (training)	-0.43	0.20	-0.82	-0.04	-0.76	0.14	4.19	1	0.041
ZI (zero inflated) intercept	-0.18	0.27	-0.71	0.35	-0.85	0.41			

[1] Some p-values are not indicated due to their limited interpretation.
[2] Continuous fixed effects were z-transformed to a mean zero and a standard deviation of one; original mean(SD) was HVR eaten: 22.22(16.45).
[3] Reference level for Condition is Assurance Game

SM References

S1. Forstmeier W., and Schielzeth, H. (2011). Cryptic multiple hypotheses testing in linear models: overestimated effect sizes and the winner's curse. Behav. Ecol. Sociobiol. *65*, 47–55. 10.1007/s00265-010-1038-5.

S2. Dobson, A.J., and Barnett, A.G. (2018). An Introduction to Generalized Linear Models (CRC Press).

S3. Field, A. (2005). Discovering statistics using SPSS: (and sex, drugs and rock'n'roll) (SAGE Publications).

S4. Fox, J., and Weisberg, S. (2019). An R Companion to Applied Regression (Sage).
